# Pollinator Behaviour on a Food-Deceptive Orchid *Calypso bulbosa* and Coflowering Species

**DOI:** 10.1155/2015/482161

**Published:** 2015-03-12

**Authors:** Juha Tuomi, Juho Lämsä, Lauri Wannas, Thomas Abeli, Anne Jäkäläniemi

**Affiliations:** ^1^Department of Biology, University of Oulu, 90014 Oulu, Finland; ^2^Department of Earth and Environmental Sciences, University of Pavia, Via S. Epifanio 14, 27100 Pavia, Italy; ^3^Oulanka Research Station, Thule Institute, University of Oulu, Liikasenvaarantie 134, 93999 Kuusamo, Finland

## Abstract

Food deception as a pollination strategy has inspired many studies over the last few decades. Pollinator deception has evolved in many orchids possibly to enhance outcrossing. Food-deceptive orchids usually have low pollinator visitation rates as compared to rewarding species. They may benefit in visitations from the presence (magnet-species hypothesis) or, alternatively, absence of coflowering rewarding species (competition hypothesis). We present data on pollinator visitations on a deceptive, terrestrial orchid *Calypso bulbosa*, a species with a single flower per plant and whose flowering period partly overlaps with rewarding, early flowering willows (*Salix* sp.) and later-flowering bilberry (*Vaccinium myrtillus*). When surveying inactive bumblebee queens on willows in cool weather, about 7% of them carried *Calypso* pollinia. Most common bumblebee species appeared to visit and thus pollinate *Calypso*. Bumblebees typically visited one to three *Calypso* flowers before flying away, providing some support for the outcrossing hypothesis. We conclude that, regarding the pollinations strategy, both magnet-species and competition hypotheses have a role in the pollination of *Calypso*, but on different spatial scales. On a large scale rewarding species are important for attracting pollinators to a given region, but on a small scale absence of competition ensures sufficient pollination rate for the deceptive orchid.

## 1. Introduction

Evolution of pollinator deception in orchids has gained considerable scientific attention [1–5]. Food deception as a pollination strategy is puzzling because, in most animal pollinated plants, resource allocation to pollinator attraction and reward is particularly important for successful sexual reproduction. In orchids, pollen is packed into pollinia that cannot be utilized by pollinators as a food resource; moreover, deceptive orchids produce no nectar. Therefore, nonrewarding orchid species usually have reduced pollinator-visitation rates when compared with rewarding species [4].

A lower visitation rate may be compensated by the fact that deceptive plants do not allocate resources to nectar production, and in orchids a single visit can remove a large amount of pollen. The avoidance of resource costs of nectar production are most likely to outweigh the reduced amount of pollination events if it is the resources and not the pollen availability that limits the seed set. However, the reverse is true in many deceptive orchids [4, 6]. A further potential advantage is an increased outcrossing rate [3]. Pollinators spend more time in foraging on a single inflorescence and visit more flowers on a single rewarding plant [7, 8]. In contrast, when a pollinator forages deceptive flowers, it usually learns after a few trials to avoid them. As a consequence, deceptive species may in a greater extent avoid inbreeding [3].

Pollinator behavior undoubtedly has a key role in the evolution of deceptive flowers [5, 9]. To receive visits from pollinators, deceptive orchids presumably take an advantage of innate, sensory, and behavioural biases of pollinators, for example, flower colour and/or odour can attract pollinators from a distance [10]. Pollinators usually gather to abundant food resources and may occasionally also visit nonrewarding plants in the vicinity of food resources. Hence, it is often assumed that deceptive orchids benefit from the rewarding neighbours that the pollinators are attracted to (“magnet-species hypothesis” [7, 11, 12]). Moreover, the mistakes made by pollinators are expected to increase with increasing similarity of flower morphology, colour, and scent between rewarding and deceptive plants (“flower-mimicry hypothesis” [5, 13–16]). In this case, deceptive species exploit innate sensory biases of pollinators, with an increasing similarity between the rewarding flower and deceptive orchid limiting the ability of pollinators learning to avoid nonrewarding flowers. On the other hand, when pollinators learn to distinguish among rewarding and deceptive flowers, rewarding species will be superior competitors and their presence can reduce visitation rates on deceptive flowers. In such a case and in contrast to the magnet-species hypothesis, deceptive species are expected to succeed best when there are no rewarding coflowering species in the close neighbourhood (competition hypothesis [7, 17]; remote habitat effect [18]).

In the present study, we investigated pollinator behavior on the deceptive orchid* Calypso bulbosa* and its coflowering rewarding species.* Calypso* is a terrestrial orchid which flowers in late spring and early summer and which is pollinated by naive bumblebee queens [10, 19–21]. Direct observations of visitations on deceptive orchids are rare because of low visitation rates. For instance, Boyden [10] reports only one direct observation of bumblebee queen visitation, and he concluded that bumblebee workers are too small to be effective pollinators for* Calypso*. Wollin [22] observed* Bombus pascuorum* visiting* Calypso*, and Alexandersson and Ågren [21] reported that they “caught queens of* Bombus hypnorum*,* B. jonellus* and* B. pratorum* with pollinia attached to the rear part of the thorax.” Boyden [10] had made similar observations with the northern American bumblebee species.

Here we report direct observations on bumblebee queens visiting* Calypso* and because of cool weather in some years, we were able to collect exceptional data of rather inactive bumblebee queens on willows and observe whether they carried* Calypso* pollinia or not. These data enable us to evaluate the importance of different pollinator species for* Calypso*. In principle, the main pollinators can either be “specialists” specifically attracted to* Calypso* or “generalists” that more or less randomly visit* Calypso* when foraging coflowering rewarding plants. Pollinator behavior on* Calypso* can also give cues whether deception enforces outcrossing (visitations on one or a few flowers only) or not (pollinators generally visit many flowers). The duration of a flower visitation, on the other hand, can reflect how experienced versus unexperienced the pollinator may be with regard to* Calypso* (a short versus long visit). Consequently, direct observations of pollinators and their behavior can provide important natural historical evidence concerning key assumptions of the hypotheses on the evolution of pollinator deception.

## 2. Materials and Methods

The study was carried out in the Oulanka National Park, Kuusamo, NE Finland (66°N, 29°E).* Calypso bulbosa* (L.) Oakes (Orchidaceae) is a small northern orchid growing in shady herb-rich old-growth forests. It has a single leaf and a flower. Pollen is packaged in two pairs of pollinia forming a pollinarium [23].* Calypso* begins to flower in late May and usually flowering is over by the end of June. At this time of year, the most important sources for nectar and pollen are early flowering willows. When willow flowering is over, bilberry (*Vaccinium myrtillus,* Ericaceae) is the most important nectar and pollen source.* Daphne mezereum* (Thymelaeaceae) is the only flowering plant in our study area that grows in the same sites and flowers at the same time as* Calypso*.* Daphne* appears to be nectarless in our study area [24].

*Calypso* is self-compatible, but self-pollination and -fertilization do not commonly occur autonomously [10, 25]. Flowering time is usually about two weeks [10]. The fruit set of* Calypso* populations in our study area varies from 20% to almost 100% between years. The average of 10 populations over three years (2003, 2006, and 2007) was 60% [26]. Hand cross-pollinations improved fruit set in most cases indicating that pollen limitation is common [21, 26]. In Sweden, *F*-statistics (*F*_IS_ = 0.283, *F*_ST_ = 0.072) indicate a high level of inbreeding (due to common self-fertilization and/or populations which are genetically substructured as a consequence of limited seed dispersal within populations) and low to moderate genetic differentiation among populations (due to common long-distance seed and/or pollen dispersal [27]).

In 2007, we established four sites of 20 × 20 m where we systematically surveyed bumblebees daily for the period 30 May–13 June. The plots were visited 1-2 times day^−1^ and all time observations were done for 30 min. The order of visitations among sites was changed daily. All observed bumblebees were identified to the species level when possible, and their behavior was recorded: flying-through in a height above or below 1 m, landing to the plot, how they moved within the plot, and visitations both on* Calypso* and/or coflowering species, especially* Daphne*. If the bumblebee landed on a flower, the duration of the visitation was estimated in addition to whether it moved to other flowers or flew away. On the plots, there were on average 49.5 ± 12.6 SD flowering* Calypso*. There were no willows on the plots or in their immediate neighbourhood (<50 m). The relative coverage of bilberry varied between 3 and 19% in the neighborhood of the study plots, but bilberry did not grow within any of the plots. Other plants are neglected here because of no visitations. In 2008–2010, further visitations were recorded as we regularly worked with* Calypso* populations in this area including the present study plots.

All visitations on* Daphne* that we observed in the study area were recorded during the study years when we were monitoring* Calypso* populations. In 2008, additional visits were daily surveyed on a sunny riverside plot (57 plants with 2–180 flowers, 22–27 May) for total of 505 min and a shady forest plot (36 plants with 2–32 flowers, 2–5 June) for 240 min. The behavior of bumblebees was recorded as above in the case of* Calypso*.

In 2007–2010, bumblebees were surveyed on flowering willows along roadsides (*Salix caprea*,* S. phylicifolia*, and* S. hastata*) and a wetland (*S. myrsinites*). In the forest, there were a few old* S. caprea*, but they were too tall for reliable observations. Most data is from the roadside sites about 100 m from our closest study plots. There are some* Calypso*s in the immediate vicinity of one willow site but not of the others. The data presented here include observations in which we could identify the species and clearly see whether they carried pollinia or not. In 2007, we also counted the number of pollinia, which was not done in the other years. The close observations were possible in cool weather (≤10°C). If too warm, as in 2009, bumblebees moved too fast to make reliable observations. In 2010, willows flowered so early that there was a mismatch with the flowering of* Calypso*; so only one individual carrying pollinia was found. Therefore, we present bumblebee data on willows only from 2007 to 2008. Every year the sites were visited once a day, until no bumblebees were found on willows. The number of individuals and the species composition varied from day to day so that it is unlikely that we repeatedly sampled the same pollinia carrying individuals. Usually, the inactive individuals were found in the morning or evening, while they flew more actively during warmer hours in the middle of the day. The distributions of bumblebee species and individuals carrying pollinia were compared by the *G* ^2^ test for the pooled data of 2007-2008.

In 2007, observations on bilberry were done 2–11 June close to the* Calypso* study plots along a path of about 200 m. In 2008–2010, first visitations on bilberry were recorded.

In Kuusamo area, 14* Bombus* (Apidae) species have been recorded [28]. Most abundant species are* B. lucorum* L.,* B. pratorum* L.,* B. jonellus* Kirby,* B. hypnorum* L.,* B. cingulatus* Whalb., and* B. pascuorum* Scop. which together make 97% of individuals. Because* B. hypnorum* and* B. cingulatus* are very similar, we made no effort to separate them in the field conditions and below they are, for simplicity, reported in the main text under* B. hypnorum*. Accordingly, no effort was made to separate* B. lucorum* from the less common, but ecologically and morphologically very similar* B. sporadicus* Nyl. or* B. cryptarum* F. The observations were made on queens only since, for instance, in 2007, the first bumblebee workers were found on 27 June when* Calypso* had already flowered. The only worker-visitation on* Calypso* was observed in mid-June 2010. It first foraged on bilberry and then visited a few* Calypso*s but no pollinaria were removed (cf. [10]).

## 3. Results

**Figure 1:**
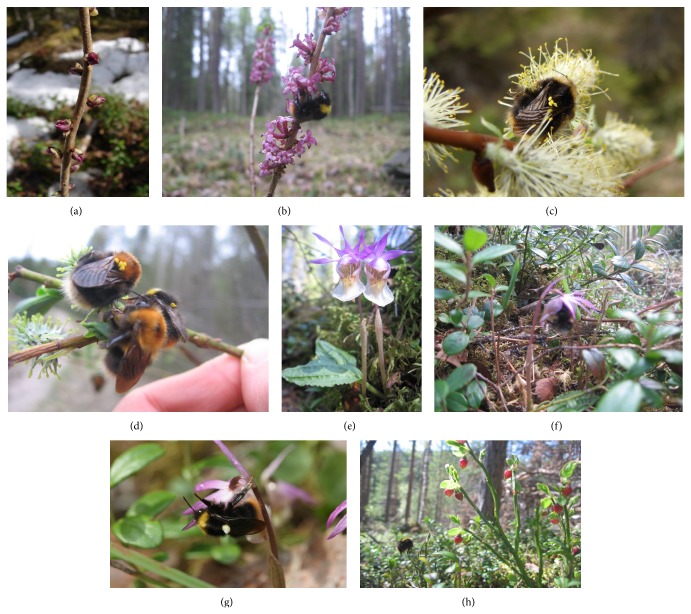
*Calypso bulbosa,* its pollinators, and coflowering species; (a) flowers of* Daphne mezereum* at a forest site, still with melting snow (May 2008, JT); (b) slowly moving* Bombus pratorum* on flowering* D. mezereum* at a forest site (May 2007, LW); (c)* B. pratorum* with several pairs of* Calypso* pollinia on willow (May 2008, JT); (d) inactive bumblebee queens on willow either with pollinia (behind:* B. pratorum* with 2 pairs of pollinia; uppermost:* B. hypnorum/cingulatus* with 5 pairs of pollinia) or without (*B. hypnorum/cingulatus* lowest) (June 2007, LW); (e) two flowering* C. bulbosa* (June 2007, LW); (f)* Bombus* sp. visiting a* Calypso* flower (2007, LW); (g)* B. pratorum* atypically walking on* C. bulbosa* flower and with a pollinarium covered by a cap (May 2010, TA); and (h)* Bombus* sp. and* Vaccinium myrtillus* at the start of flowering (June 2007, LW). Photos by Thomas Abeli (TA), Juha Tuomi (JT), and Lauri Wannas (LW) in the Oulanka National Park, Kuusamo, Finland.

**Figure 2:**
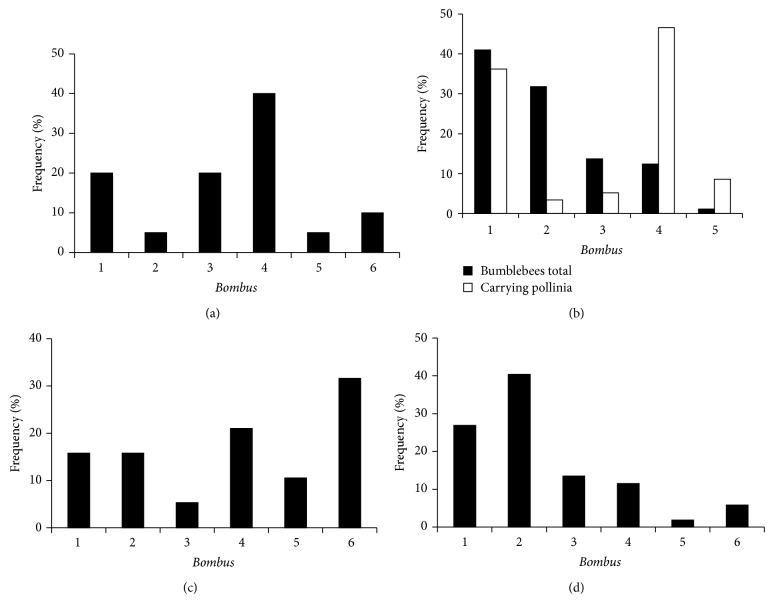
Relative abundance (%) of bumblebee species on (a)* Daphne mezereum* in 2007–2010, *N* = 20; (b)* Salix* spp. in 2007-2008 including total number of bumblebees (black bars, *N* = 812) and individuals carrying* Calypso* pollinia (white bars, *N* = 58), (c)* Calypso bulbosa* in 2007–2010, *N* = 19, and (d)* Vaccinium myrtillus* in 2007, *N* = 58. Species: 1 =* Bombus hypnorum/cingulatus*, 2 =* B. jonellus*, 3 =* B. lucorum*, 4 =* B. pratorum*, 5 =* B. pascuorum*, and 6 =* Bombus* sp.

**Figure 3:**
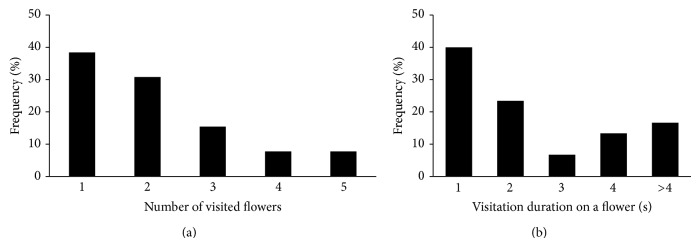
Visitations on* Calypso bulbosa* flowers by bumblebee queens within study plots in 2007. Frequency distribution for (a) number of visited flowers (*N* = 13 bumblebees) and (b) visitation duration in seconds on a single flower (*N* = 30 flowers).

We first describe the phenological window of* Calypso* flowering in relation to the coflowering, rewarding species (Table 1). Then we report bumblebee visitations and behavior on the plants following the phenological order from* Daphne* to willow and* Calypso* and finally bilberry (Figures 1–3).

### 3.1. Flowering Phenology

The start of flowering of* Calypso* varied from a year to the next and with it varied the phenological relations with the most important nectar sources of bumblebees and coflowering species of* Calypso* (Table 1). First, there is an overlap of 2–6 days with flowering willows. Seconds, there is a phenological window of 2–8 days after the flowering of willows and before bilberry starts to flower, in which* Calypso* is the only flowering species. These two periods make together 6–13 days before bilberry flowering. Third, the end of* Calypso* flowering overlaps with bilberry flowering for about 1-2 weeks.

### 3.2. Low Visitation Rates on* Daphne*

In sunny riversides,* Daphne* can start to flower already in early May on spots where snow has smelted. In forest, it begins to flower few days before* Calypso* (Figure 1(a)). After some days flowers become pale, and usually fresh flowers are rare at the time of the peak flowering of* Calypso*. Bumblebees were most often found on* Daphne* in late May (*N* = 20, 18 May–2 June, 2007–2010).* Bombus pratorum* (40%),* B. hypnorum* (20%), and* B. lucorum* (20%) were the most frequent species (Figure 2(a)). Some bumblebees observed on* Daphne* also visited or had earlier visited* Calypso*. In 2007, one* B. pratorum* carried pollinia. In 2008,* B. pratorum* was observed to fly from* Daphne* to a* Calypso* flower. In 2009, an unidentified* Bombus* approached first* Daphne* but suddenly changed direction to* Calypso* and removed the pollinia.

Bumblebees were either actively foraging (45%) or completely inactive or slowly walking on the inflorescences (55%, Figure 1(b)). Actively foraging individuals in most cases visited one plant only (80%, *N* = 10 observations), and on average 8.5 ± 5.9 SD flowers per plant (*N* = 6 plants). In the riverside survey plot in late May (2008), 8 bumblebees were seen of which 50% landed on a plant. This indicates a low visitation rate of one visit per plot in 2 h and per plant in 120 h. In the forest survey plot in early June, the rate appeared to be even lower because no bumblebees were seen in spite of 6 h monitoring.

### 3.3. Pollinia Carrying Bumblebees on Willow

In cool weather bumblebees remained on the willows and, during their stay, it was easy to identify the species and check whether they carried* Calypso* pollinia (Figures 1(c) and 1(d)). In 2007-2008, we observed 812 queens of which 7.1% carried pollinia (Table 1). The distribution of pollinia carrying individuals among the species differed from the abundance distribution of all observed bumblebees (Figure 2(b), *G* ^2^ = 81.5, df = 4, *P* < 0.001).

Pollinia were most frequently found on* B. pratorum* (46.6% of all pollinia observations in 2007-2008). This species was moderately abundant (12.4% of all bumblebees) and about 26.7% of 111 individuals carried pollinia.* B. hypnorum* was the most common species representing 41% of all bumblebees. Only 6.3% of 333* B. hypnorum* carried pollinia, but still about 36.2% of pollinia were found on this species.* B. pascuorum* was relatively rare (1.1% of all bumblebees), but 55.6% of them (*N* = 9) carried pollinia. As the result, 8.6% of pollinia were found on this species.* B. jonellus* and* B. lucorum* are common species but they rarely carried pollinia (0.8 and 2.7% of 258 and 111 individuals, resp.).

On most cases checked on bumblebees in 2007, there were two pairs of pollinia (23 cases) or less (4) or more (6 cases). In four cases there were 4 pairs, in one case 5 pairs, and in one case 7 pairs. Thus at least 18.2% of pollinia carrying bumblebee queens had visited several* Calypso* flowers (see also Figures 1(c) and 1(d)). In 2009, one bumblebee fell down from a willow and flew to a* Calypso* growing few meters away.

### 3.4. Direct Observations of Visitations on* Calypso*

All 19 visitations were observed between 24 May and 15 June 2007–2010. At the time of visitations, temperature was always above 14°C. Among the species observed on the orchid,* B. pratorum* was the most common and* B. lucorum* the rarest species (Figure 2(c)).

On the four survey plots in 2007, we observed a total of 64 bumblebee queens of which 10 (16%) visited flowering* Calypso*. In 22 cases bumblebees landed on survey plots, and in 14 cases they flew across the site at a height below 1 m. On the* Calypso* survey plots we observed a bumblebee only once on* Daphne* but never on any other coflowering plant.

Visitations on* Calypso* in the survey plots were distributed as follows:* B. lucorum* (1 of 16 landing on the plot or flying <1 m),* B. jonellus* (2 of 4),* B. hypnorum* (2 of 10),* B. pratorum* (3 of 6), and* Bombus* sp. (2 of 10). In addition to these, in 2007–2010, we observed 9 visits by* B. hypnorum* (1),* B. jonellus* (1),* B. pratorum* (1),* B. pascuorum* (2), and* Bombus* sp. (4 visits). In 2007 at the survey plots, bumblebees visited usually 1–3 flowers (Figure 3(a)).

Most often (63.4% of cases) visitation on a flower lasted 1-2 seconds or, alternatively, ≥4 seconds (29.9%, Figure 3(b)). Visitations can roughly be classified into four groups: one flower (6 observations), shortly 2-3 flowers with ≤2 seconds per flower (4), 2–4 flowers with several seconds in the first and briefly in the other(s) (4), and many flowers with variable times per flower (1). After the visitation(s), they flew away from the site.

During a typical visitation, bumblebees landed on the labellum and pushed into the flower trying to reach to the spur (Figure 1(f)). As an example of atypical visitation,* B. pratorum* was observed in 2010 to move very slowly on a flower (for about 2 min). It removed the pollinarium with the cap still attached (Figure 1(g)). Then it slowly walked to a second flower (2 min), which was not pollinated, and a third one from which it fell down to the ground.

### 3.5. Bumblebees on* V. myrtillus*

In 2007, five* Bombus* species were observed on bilberry (Figure 2(d)):* B. jonellus* (21 observations),* B. hypnorum* (14),* B. lucorum* (7),* B. pratorum* (6),* B. pascuorum* (1), and* Bombus* sp. (3). Pollinia were observed on 3 individuals of* B. pratorum* and one* B. lucorum*. Bilberry is “buzz pollinated.” Therefore it is notable that, in June 2007 when bilberry was already flowering, a bumblebee landed on a* Calypso* flower and performed buzz-pollination behaviour by hanging down from the labellum, in the way bumblebees usually do with bell-formed flowers of bilberry (Figure 1(h)).

## 4. Discussion

All abundant bumblebee species visited* Calypso* as well as coflowering species. The pollinia observed on bumblebee queens found on willows suggest that* B. pratorum* and* B. hypnorum*/*cingulatus* are the most important visitors of* C. bulbosa*. These two species represented over 80% of all bumblebees carrying pollinia.* B. hypnorum* is only moderately attracted to* Calypso*, but because of its high abundance, up to 35% of pollinia were found on this species.* B. pratorum* and* B. pascuorum* were in a greater extent attracted to* C. bulbosa*, but the latter is less abundant in our study area. It is notable that* B. lucorum* and* B. jonellus*, which are common on bilberry, rather rarely carried pollinia in relation to their abundance on willows.

Willows and bilberry are potential magnet-species provided that their flowering overlaps with* C. bulbosa* flowering. Overlap can vary greatly from a year to the next depending on weather conditions in May and June.

In spite of the frequent cooccurrence of flowering* D. mezereum* and* C. bulbosa* in our study area, we have not observed any consistent or strong magnet effect of* Daphne*. This is consistent with the fact that* Daphne* is also a nonrewarding species in Scandinavia [24]. Still, it might provide some pollen reward and the flowers elicit pheromone-like scent (linalool [29]). According to Borg-Karlson et al. [29], insects might visit* Daphne* flowers because linalool is often associated with nectar-rich plants such as willows. Indeed, occasionally a number of bumblebees can be attracted to sunny* D. mezereum* sites, especially before willow flowering, and shifts from* Daphne* to* Calypso* do occur. However, our estimates of the visitation rate on* D. mezereum* are very low, and bumblebees do not very persistently forage on its flowers. In most cases, they visited one plant and then flew away. Furthermore, the fruit set of* C. bulbosa* seem not to depend on the presence of* Daphne*, which was excluded as a significant explanatory variable in the statistical tests by Abeli et al. [26].

The often rather inactive behaviour of the queens on* D. mezereum *and* Salix* spp. indicates that, during the cool weather, they tend to stay on or close to the expected nectar/pollen source. It is energetically expensive to fly from a source to the next, because flight muscles have to warm up to the minimum temperature, about 30°C, required for flight [30, 31]. It is obvious that willows are the main target for foraging bumblebees. Energy gained from willows may help them to regulate thoracic temperature which increases with sugar concentrations [32]. With low energetic gain, bumblebees let thoracic temperature drop as they slowly walk on an inflorescence. Before takeoff thoracic temperature must be elevated by shivering which takes several minutes (6 to 15 min at air temperatures of 13 to 6°C [30]). When energetic gain is greater, thoracic temperature is maintained for flight readiness and foraging movements are quicker [33, 34]. In contrast, they do not gain nectar from* D. mezereum*. Therefore, after landing on* Daphne* in cool weather, they may be forced to stay there for hours in the wait for a sunfleck or warmer weather. When directing from* D. mezereum *to willows they may also visit* C. bulbosa.*

Although willows undoubtedly are the most important source of food for the naive queens, they may have a rather limited role as a magnet-species. First, they do not frequently cooccur in the same sites of* C. bulbosa*. Usually they grow in rather open habitats in road, river, and field sides and to some extent also in wetlands. In our study area, the distance from* C. bulbosa* to the closest willows is usually 50 m to hundreds of metres, and only exceptionally they grow close to each other. Although we have observed a direct shift from a willow to the orchid in its immediate neighbourhood, the most frequent visitations on* C. bulbosa* were by bumblebees approaching the site from a longer distance. Second, the overlap with the earliest flowering individuals in* C. bulbosa* populations is only a few days and highly variable between years. Therefore, it is not surprising that the magnet-species effect of willows has been found in some years only. Alexandersson and Ågren [21] found in Sweden that pollinia removal was positively related to the density of* Salix caprea* (ranging from 0 to 0.79 ha^−1^) in one of the three study years, which gives some support for the magnet-species effect.

When willows do not flower anymore, bumblebees may most often approach* C. bulbosa* flowers when seeking for alternative nectar sources or a nest site. It is easy to imagine that* Calypso* flowers may attract them, and they can visit one or a few flowers before flying away from the site. It is notable that there is a window up to a week in June between the flowering of willows and bilberry and it often coincides with the peak flowering of* C. bulbosa.*

Later in June, bilberry can have some influence, especially for late-flowering individuals of* C. bulbosa.* Usually, bilberry is very abundant everywhere around the* Calypso* sites, but rarely within the sites. We do not have evidence for any strong magnet effect of bilberry, because rather few bumblebees on bilberry carried pollinia. In addition, their pollination mechanisms are so different that pollinators primarily foraging on bilberry may not always behave appropriately on* C. bulbosa*, as proved by our observation of a buzz-pollination attempt. There is an interesting parallelism to the Northern America where* Dodecatheon* species are potential model species for* C. bulbosa*, but Boyden [10] excluded this possibility because their odour is different and pollination requires different pollinator behavior.* Dodecatheon* requires buzz-pollination behaviour as does bilberry.

The behaviour of the pollinators on* C. bulbosa* is consistent with the hypothesis that increased outcrossing is one of the benefits of flower deception [2]. In rewarding species, pollinators often move very short distances within a single plant or between neighbouring plants, for example, in bilberry [35].* Calypso* has only a flower, but individual plants often occur in smaller or larger clusters, which may consist of close relatives. If pollinators most often visit 1–3 flowers and then fly away, inbreeding is unlikely. However, sometimes pollinators behave differently and they may visit several flowers before departure. In such cases inbreeding can take place, as suggested by Alexandersson and Ågren [27]. However, this may be quite rare because the cap covering the pollinarium may prohibit pollen transfer as long as it remains attached (Figure 1(g); for discussion, see [10, 26]).

Population structure is important also because the initial attraction of pollinators may increase with plant density or because a group of plants may be more attractive than a singly growing plant [4, 36]. This positive density dependence which arises from sensory biases of the pollinators is counterbalanced by their learned aversion of nonrewarding plants. Bumblebees can rapidly learn to avoid deceptive plants, after as few as 2–6 visits [17, 37]. Assumedly, the aversion learning would be fastest when the density of nonrewarding species is high, and hence negative density dependence of reproductive performance is often expected in deceptive orchids [38, 39].

Because flower visitation time declined with successive visitations at the same site, this indicates a learning behaviour where the bees generalized the rewardlessness over* Calypso* flowers in the close vicinity of the visited ones. The variation in the number of visited flowers indicates that some bumblebees are more experienced than others and make the generalization faster than the less experienced individuals. Alternatively, individual bumblebees may innately differ in their learning behaviour. Similar variability in the individual behaviour is observed in laboratory among workers of* Bombus terrestris* originating from the same nest (JL, unpublished). It is reasonable to assume that an individual pollinator has to visit* C. bulbosa* in several sites and perhaps in different times before it is able to generalize rewardless on the typical features of* Calypso* flowers. The number of visitations needed for the development of the aversion may depend on both the abundance of the rewardless species itself and the abundance of alternative, rewarding plants. The rewardless species may succeed best when it is rather rare and rewarding plants are not too common. This would suggest that the phenological window between early (willows) and later (bilberry) flowering nectar sources of bumblebee queens might be important for the reproductive success of* C. bulbosa.*

Consequently, both the magnet-species hypothesis (rewarding flowers have to be in the range of pollinator home range) and the competition or remote land hypothesis (no or few rewarding plants in the immediate vicinity) might influence* Calypso* pollination. We suggest that these two hypotheses could in fact work simultaneously but at different spatial scales. The magnet mechanisms could work in the larger scale by increasing pollinator density with a given area, but inside the area there has to be heterogeneity in the distribution of rewarding and deceptive flowers and sites where rewarding flowers are scarce. Thus individual rewardless plants could locally succeed best when their immediate vicinity is free from other rewardless as well as rewarding competitors.

We thus hope that our purely observational study would encourage for more rigorous tests that incorporate spatial and temporal heterogeneity in the structure of plant populations and communities as explanatory variables of pollination success in deceptive orchids.

## Figures and Tables

**Table 1:** The phenological window of *Calypso bulbosa* flowering in relation to the flowering of the most important nectar sources, early flowering willows (*Salix*) and later-flowering bilberry (*Vaccinium*). Overlap of *C. bulbosa *flowering with the coflowering species is given in number of days. The last column gives total number of *Bombus* spp. on *Salix *spp. and number of individuals carrying pollinia.

Year	First flowering *C. bulbosa *	Overlap with *Salix *spp.	Days between *Salix* and *Vaccinium *	No overlap with* Vaccinium *	Overlap with* Vaccinium *	*Bombus* spp. on *Salix* spp. (total/pollinia)
2007	<28 May	>4	2	6	≥11	469/33
2008	2 June	6	5	11	≥14	357/25
2009	24 May	6	7	13	≥8	35/0
2010	21 May	2	8	10	≥15	27/1
